# Assessment of structural lesions, synovitis and bone marrow lesions in erosive hand osteoarthritis on MRI (0.3T) compared to the radiographic anatomical Verbruggen-Veys score

**DOI:** 10.1371/journal.pone.0234972

**Published:** 2020-06-23

**Authors:** Edem Allado, Ruth Wittoek, Stephanie Ferrero, Eliane Albuisson, Isabelle Chary-Valckenaere, Christian Roux, Damien Loeuille

**Affiliations:** 1 Department Rheumatology, University Hospital of Nancy, Nancy, France; 2 Department of Pulmonary Function Testing and Exercise Physiology, University Hospital of Nancy, Nancy, France; 3 Development, Adaptation and Disadvantage, Cardiorespiratory regulations and motor control (EA 3450 DevAH), University of Lorraine, Nancy, France; 4 Department of Rheumatology, Faculty of Medicine and Health Sciences, Laboratory for Molecular Immunology and Inflammation, Ghent University, Ghent, Belgium; 5 Department Rheumatology, University of Cote d’Azur, Nice Hospital, Laboratory LAMHESS, EA6312, IBV CNRS IMR INSERM UNS, Nice, France; 6 Pôle S2R, PARC, University Hospital of Nancy, Nancy, France; 7 InSciDens, University of Lorraine, Nancy, France; 8 CNRS, Institute Elie Cartan de Lorraine, UMR, Nancy, France; Monash University, AUSTRALIA

## Abstract

**Objective:**

To evaluate prevalence of structural lesions, synovitis and bone marrow lesions (BMLs) on MRI performed with a 0.3T imaging system in patients with erosive hand osteoarthritis (EHOA) and to compare them to the anatomic radiographic Verbruggen-Veys score (VV).

**Design:**

For this Cross-sectional study, fifty-five EHOA patients were studied with 0.3T contrast-enhanced MRI and radiography (RX) of their dominant hand. Structural lesions were scored according to the OMERACT Hand Osteoarthritis MRI Scoring System as follows: osteophytes and erosions were graded from 0 to 3. On joint destruction lesion synovitis and BMLs were graded from 0 to 1. And on MRI, we evaluated the presence of several structural features: N: normal, O: osteophytic lesions, E: erosive lesions, E/O: osteophytic and erosive lesions and D: joint destruction. RX was scored according to the VV system. Relations between MRI features and VV stages were analysed.

**Results:**

MRI identified more structural lesions than RX (77.3% versus 74.8%) and particularly more erosive lesions (E or E/O) than VV Phase E (33.5% versus 20.2%). E/O and D were mostly found on MRI. Synovitis and BMLs were significantly associated with E/O and D with the following odds ratios (ORs): 8.4 (95% CI 1.8–13.6); OR: 13.7 (95% CI 2.9–21.0); OR: 15.7 (95% CI 3.2–23.5); OR: 38.5 (95% CI 9.5–57.0), respectively.

**Conclusion:**

MRI 0.3T appears completely relevant for EHOA lesion analysis. First, MRI shows more erosive lesions than RX in EHOA; second, it allows for the analysis of synovitis and BMLs to be associated with more specific structural MRI features (E/O and D).

## Introduction

Hand osteoarthritis (HOA) is a symptomatic disease associated with a functional disability of the same level as that in rheumatoid arthritis [1,2]. Flares with diurnal pain are present in more severe clinical forms, especially in females with erosive hand osteoarthritis (EHOA) on radiography (RX). The major predictors of HOA are advanced age, female sex, obesity and the presence of genetic factors [3,4]. To date, HOA treatment is based on pharmacological and non-pharmacological measures, and recent recommendations have been published [5–7]. There is still no recommendation for the use of conventional or biological disease-modifying antirheumatic drugs (e.g., Plaquenil, Methotrexate, Etanercept, Adalinumab or anti-interleukin-1β), as none of these treatments demonstrated any clinical or structural efficacies in HOA [8]

In clinical practice, RX allows the diagnosis of HOA to be established and permits structural damage to be followed over time [9]. Composite scoring system or several scores (Kellgren & Lawrence (K&L), Verbruggen–Veys score (VV), Kallman and OARSI) have been demonstrated to accurately evaluate HOA lesions with excellent intra- and inter-reader reproducibility [10–15]. The K&L system is a score based on joint space narrowing (JSN) and the presence of osteophytes, periarticular osicles and small pseudocystic areas. Kallmann et al. added lateral deformity and cortical collapse to the K&L criteria [11]. Finally, the OARSI atlas defines for the first time the terms of erosion and considers all other RX features previously mentioned. However, these scores are moderately high sensitive to change, with a standardized response mean (SRM) calculated to be 0.3 [16].

In comparison to these scores, in which each RX HOA feature is individually scored, the VV score defines HOA radiographic features according to a more qualitative approach using anatomical changes that are categorized on a 5-grade scale: normal joint "N", stationary phase "S", loss of joint space "J", erosive phase "E" and finally the remodelling phase "R".

Ultrasound and MRI provide additional information for the early diagnosis of HOA and are able to predict RX progression in HOA [17–19]. The ability of MRI to demonstrate structural lesions (osteophytes, erosions, joint narrowing, osteosclerosis) and inflammatory lesions (synovitis) and bone marrow lesions (BMLs) not visible on RX allows for a better understanding of the pathophysiological mechanisms leading to joint destruction [20–22]. Compared to RX, MRI is also more efficient at detecting structural lesions [23]. Recently, subluxations, severe joint narrowing, osteophytes, synovitis and BMLs were identified as risk factors of progression in HOA patients who underwent MRI during follow-up [19].

Until now, MRI HOA characteristics were compared to the RX scoring system based on the K&L and/or OARSI scores [21–26]. To the best of our knowledge, no study has compared structural features and their associations in patients with EHOA on a 0.3 T MRI to the anatomical VV score in which RX lesions are presented as a more “global joint assessment”.

Each synovitis, BMLs and structural elementary lesion was scored to be either a binary for synovitis, BMLs, joint bone destruction and either four levels of severity for osteophytes and erosions. Thereafter, each joint was classified into five features of structural lesions observed in EHOA: normal (N), osteophytic (O), erosive (E), erosive and osteophytic (E/O) and complete destruction (D). This global joint assessment has been well evaluated in VV score. The purpose of this study was to evaluate the prevalence and severity of hand structural lesions (osteophytes, erosions, joint destruction), synovitis and BMLs on a 0.3T MR imager and structural damage on RX using the VV score. Second, we evaluated the associations between synovitis, BMLs observed on MRI and structural lesions observed on both MRI and RX.

## Method

### Patients

Patients with EHOA were included in the ADEM cohort in a double blind, randomized, prospective monocentric interventional study promoted by the Department of Rheumatology of Nice (clinical trial NCT01068405) from May 2010 to April 2013. This trial aims to compare the efficacy of methotrexate for the treatment of structural lesions after 12 months and the clinical symptoms at 3 months in patients with EHOA.

The inclusion criteria for the clinical trial were as follows: patients aged between 45 and 85 years, patients suffering from EHOA in the proximal interphalangeal (PIP) and distal interphalangeal (DIP) joints for more than three months; response to the ACR criteria for HOA; and a visual analogic scale (VAS) score greater than 40 mm. Patient in whom failure of symptomatic slow-acting drugs for OA (SYSADOA) occurred should have stopped their treatment at least 3 months before inclusion. Patients who received injections of hyaluronic acid in hand joints in the last 6 months and cortisone derivatives in the previous 3 months were excluded. Finally, treatment by NSAIDs or level 1 antalgics should not have been modified in the past 4 weeks before inclusion. Reliable contraception was taken throughout the duration of the trial and continued for 3 months after the cessation of treatment in women of childbearing age and for 5 months in men. Exclusion criteria were as follows: pregnant or breastfeeding women and patients with psoriasis, inflammatory rheumatic diseases (RA, SpA, PSoA, and connective tissue diseases), crystal arthropathies, severe hepatic or renal insufficiency, contraindications for the use of methotrexate, the presence of joint prosthesis in one of the joints of the hand and, finally, contraindication to MRI. To be included in this study, patients had to present with at least one eroded joint among the PIP or PID joints on bilateral hand RX.

### Demographic and clinical data

The patients underwent a physical examination of the hands for PIPs, DIPs, metacarpophalangeal joints (MCPs) for a total of 14 joints. Swollen nodes and tender joints were counted (absent or present), and the visual analogic scale (VAS) was used to score hand pain (0–100 mm); the VAS was for the evaluator’s global disease activity and the patient global disease activity.

All patients were asked to fill out two questionnaires: the Functional Index for Hand Osteoarthritis (FIHOA) and Cochin Hand Functional Scale. The former, which consists of questions concerning three domains (pain, stiffness, and physical function), can result in a score from 0 (none) to 30 (extreme); individual items are rated on a 4-point scale from 0 (possible without difficulty) to 3 (impossible) [27]. The purpose of the self-reported Cochin Hand Functional Scale is to measure functional ability in the hand. There are 18 items in 5 subscales: kitchen (8 items), dressing (2 items), hygiene (2 items), office (2 items), and other (4 items).

### Conventional hand radiography

Hand RX was performed for all patients at the baseline. Blind evaluation of the radiographs was performed by a senior and junior independent reader (SF and RW). A comparison of the two readings was performed on erosive joints (VV scoring system). The concordance between evaluators was 82%. Only the senior reading (RW) was considered for the analysis. The anatomical VV score was determined for both hands. This score is based on a more qualitative approach to RX features in HOA defined on 5-phase ordinal data: normal joints are classified as phase "N", stationary phase "S" (classic appearance of HOA with more osteophytic feature), loss of joint space is classified as phase "J" (disappearance of the joint space), erosive phase "E" (concurrently or just after J phase, with erosion of the subchondral bone), and a remodelling phase "R" (new irregular sclerotic subchondral plate formation, huge osteophytes are formed during this phase).

### MRI of the hand

The proximal and distal interphalangeal regions from the 2nd to 5th digits of the dominant hand were evaluated on a 0.3 Tesla MR imager (C-Scan Esaote Biomedica, Genoa, Italy) at the baseline for each patient. For each sequence, axial and coronal planes were used with the following sequences: spin Echo 3DTI (Repetition Time (TR) 35, echo time (TE) 16, Field of view (FOV) 160x160, Matrix 192x192), STIR (Repetition Time (TR) 1460 ms, Echo Time (TE) 26 ms, Field of view (FOV) 190x190 mm, matrix 192x168, slice thickness 3 mm), high GE 16 T1 before and after gadolinium injection (Repetition Time (TR) 500, Echo Time (TE) 16, Field of view (FOV) 160x160, Matrix 192x192, slice thickness: 2 mm); they were selected according to the OMERACT protocol recommendations^16^.

MRI analysis derived from the OMERACT Hand Osteoarthritis MRI Scoring System model was performed by senior and junior readers (DL, EA) blinded to the clinical data. Related to the low spatial resolution (0.99 x 1.1 mm^2^) with a slice thickness of 3 mm, we decided to score MRI features in the following manner: osteophytes (grade 0–3, distal or proximal) and erosions (grade 0–3, distal/proximal). Synovitis, BMLs and joint destruction were scored according to a binary analysis (absent/present). Intra-reader and inter-reader reliabilities were tested on a set of 20 EHOA patients representative of the study population chosen at random.

**Fig 1:**
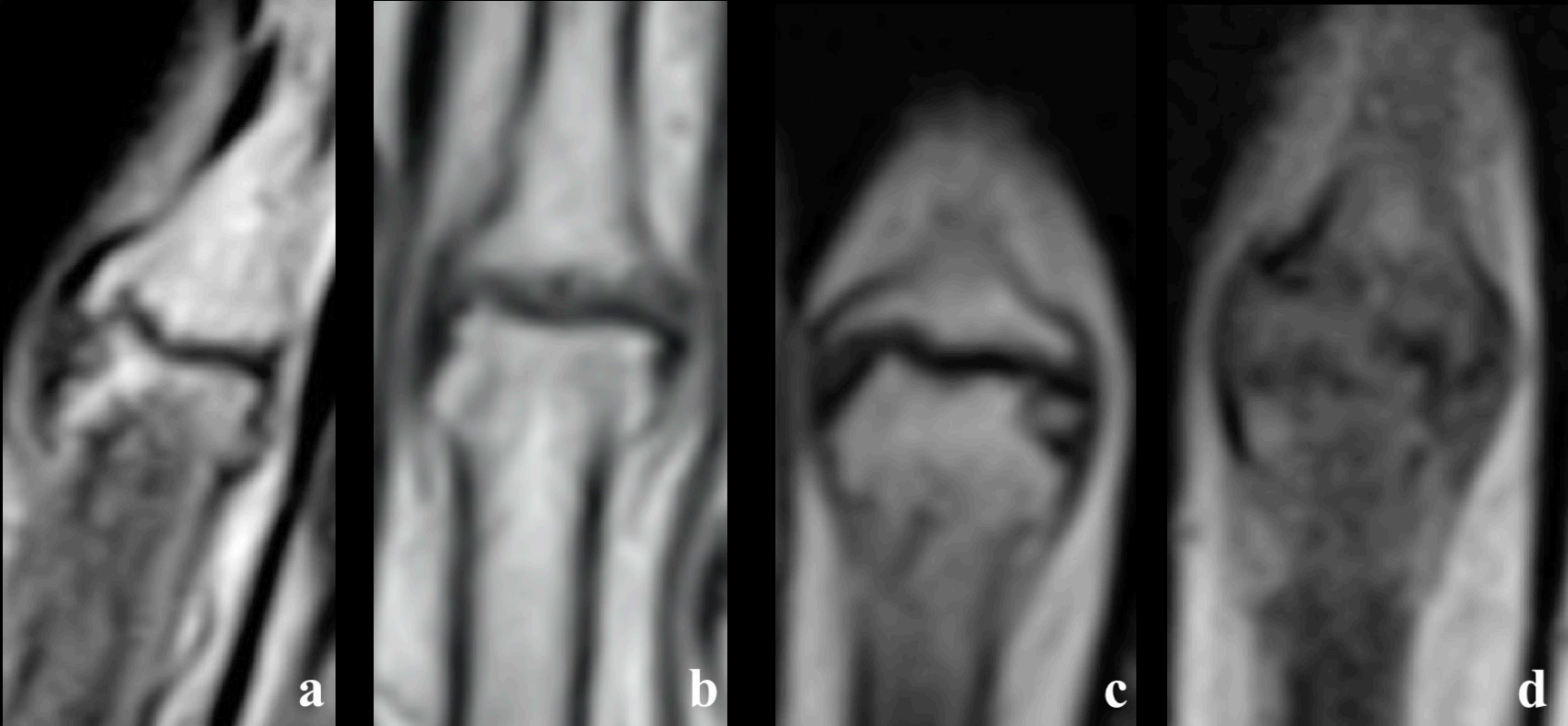
MRI joint structural features. a.: osteophytic feature (O) b.: erosive feature (E) c.: erosive and osteophytic lesion feature (E/O) d.: complete articular destruction (D).

We evaluated all structural MRI features observed in EHOA and some associations because erosion and osteophytes may be observed on the same joint. Thus, five MRI joint structural features were evaluated: normal feature (N), osteophytic feature (O), erosive feature (E), erosive and osteophytic lesion feature (E/O) and feature with complete articular destruction (D) (Fig 1). It’s obvious that normal joint may present BMLs or synovitis. but these may be also observed in joint without structural lesions.

### Statistical analysis

Both descriptive and comparative analyses were conducted according to the nature and the distribution of the variables. Qualitative variables are described with frequencies and percentages; quantitative variables are reported as the mean ± SD (standard deviation) or as the median and interquartile range (IQR). The chi-square test, was used for the analysis. To analyse the intra-reader and inter-reader reliability, we used Cohen’s kappa test according to Landis and Koch [28]. Analyses were performed using IBM SPSS Statistics V.23, and p values <0.05 were considered statistically significant.

### Ethical considerations

All data used were obtained from medical records of the cohort of patients. This study is registered with the Information Technology and Freedoms Commission for the University Hospital of Nice (09–029 at 18/01/2010) and was designed in accordance with the general ethical principles outlined in the Declaration of Helsinki. All patients gave their informed consent for the use of their medical data during the time period they received medical care at the University Hospital of Nice.

## Results

### Demographic data and clinical characteristics

Among the 64 patients included in the clinical trial, 55 patients were examined on both imaging modalities (RX and MRI) and were selected for this study according to the inclusion criteria. The median age was 67 (62–72) years. The included patients suffered from severe pain and dysfunction: the mean VAS score for pain was 64.5 mm (+/-13.7); the mean evaluator’s global disease activity VAS score was 63.3 mm (+/-12.3), and the mean patient global disease activity VAS score was 59.7 mm (+/-14.6). The median 14-joint counts for tenderness, nodes and swollen joints were 5 (3–8), 4 (3–5) and 3 (2–3), respectively. The median Functional Index for Hand Osteoarthritis (0–30) score and the average Cochin Hand Functional Scale (0–90) score were calculated to be 12 (7–17) and 26 (10.75–44), respectively (Table 1).

**Table 1:** Baseline demographic and clinical characteristics (n = 55).

Women	52 (94.5%)
Age, years	65.7 (5.7)
Body mass index, kg/m^2^	24.3 (3.0)
Visual analogic scale for pain [0–100] (mm)	64.5 (13.7)
Evaluator’s global disease activity (mm)	63.3 (12.3)
Patient global disease activity (mm)	59.7 (14.6)
Tender joint count of dominant hand [0–14])	5 (3–8)
Node joint count of dominant hand [0–14])	4 (3–5)
Swollen joint count of dominant hand [0–14])	3 (2–3)
Cochin Hand Functional Scale (0–90)	29.5 (18.3)
Functional index for hand osteoarthritis (FIHOA) (0–30)	12.3 (5.2)
Patients with one or more abnormal interphalangeal joints according to MRI features (n = 55)	
• Osteophytes (≥1 joint)	54 (98%)
• Erosion damage (≥1 joint)	53 (96%)
• Bone marrow lesions (≥1 joint)	34 (62%)
• Synovitis (≥1 joint)	20 (36%)

Data are presented as n (%) for dichotomous variables and as the mean (SD) or median (interquartile range: IQR) for continuous demographic variables and patient-reported outcomes according to the distribution.

For VV score: N–Normal joint; S–Stationary phase, J—Joint space phase, E–Erosive phase R–Remodelling phase.

### MRI reproducibility and reliability

For the MRI score, a blind evaluation of inter-reader (DL—EA) and intra-reader reproducibility (EA) was performed using Cohen's kappa on a sample of 20 patients (160 joints) with a delay of 6 weeks between the two readings. Intra-reader reproducibility values for erosion, osteophytes and destruction were 0.75 (95% CI 0.68 to 0.82), 0.77 (95% CI 0.70 to 0.83) and 0.93 (95% CI 0.84 to 1.00), respectively. Intra-reader reproducibility for synovitis and BMLs was excellent, calculated at 0.91 (95% CI 0.82 to 1.00) and 0.94 (95% CI 0.84 to 1.00), respectively.

Inter-reader reproducibility values for erosion, osteophytes and destruction were 0.76 (95% CI 0.70 to 0.83), 0.78 (95% CI 0.71 to 0.84) and 0.94 (95% CI 0.84 to 1.00), respectively. Inter-reader reproducibility valued for synovitis and BML were excellent, calculated at 0.94 (95% CI 0.85 to 1.00) and 0.96 (95% CI 0.87 to 1.00), respectively.

Inter-reader reproducibility and intra-reader reproducibility values for MRI structural features were 0.77 (95% CI 0.70 to 0.83) and 0.83 (95% CI 0.76 to 0.88), respectively.

### MRI and Verbruggen-Veys radiographic scoring system results

- VV radiographic scoring system: of 440 joints, 109 (25%) were considered normal (N), 168 (38%) were in the stationary phase (S), 15 (3%) were in phase J, 96 (22%) were in the erosive phase (E) and 52 (12%) were in the remodelling phase (R) (Table 1). The prevalence of osteoarthritic structural lesions was relatively higher in both DIP 2 and DIP 3 joints (n = 46; 84%), followed by DIP 4 and DIP 5 joints (n = 44; 80%) (Table 2).
- MRI scoring system: among the 361 joints available on MRI, 279 joints (77%) presented at least one osteoarthritic joint feature (osteophytes, erosions, BMLs, synovitis and destruction). Osteophytes and erosions were present in 266 (74%) and 206 (57%) patients, respectively. Both lesions were simultaneously present in 196 patients (54%). Concerning BMLs and synovitis, the prevalence were 14% and 8%, respectively (Table 3).

**Table 2:** The prevalence of Verbruggen-Veys radiographic scoring system features in interphalangeal joints of 2th–5th fingers.

	2nd PIP n (%)	3th PIP n (%)	4th PIP n (%)	5th PIP n (%)	2nd DIP n (%)	3th DIP n (%)	4th DIP n (%)	5th DIP n (%)	TOTAL
**VerbrugenVeys score**	**55**	**55**	**55**	**55**	**55**	**55**	**55**	**55**	**n = 440**
Normal phase (N)	20 (36)	16 (29)	18 (33)	15 (27)	9 (16)	9 (16)	11 (20)	11 (20)	**109 (25)**
Abnormal phase	35 (64)	39 (71)	37 (67)	40 (73)	46 (84)	46 (84)	44 (80)	44 (80)	**331 (75)**
Phase (S)	23 (42)	23 (42)	28 (51)	24 (44)	18 (33)	14 (25)	21 (38)	17 (31)	**168 (38)**
Phase (J)	1 (2)	2 (4)	1 (2)	1 (2)	1 (2)	1 (2)	4 (7)	4 (7)	**15 (3)**
Phase (E)	6 (11)	10 (18)	4 (7)	10 (18)	17 (31)	19 (35)	15 (27)	15 (27)	**96 (22)**
Phase (R)	5 (9)	4 (7)	4 (7)	5 (9)	10 (18)	12 (22)	4 (7)	8 (15)	**52 (12)**

The number (%) of affected joints is presented

For VV score: N–Normal joint; S–Stationary phase, J—Joint space phase, E–Erosive phase R–Remodelling phase.

**Table 3:** The prevalence of MRI in interphalangeal joints of 2th–5th fingers.

	2nd PIP n (%)	3th PIP n (%)	4th PIP n (%)	5th PIP n (%)	2nd DIP n (%)	3th DIP n (%)	4th DIP n (%)	5th DIP n (%)	TOTAL
MRI (Total)	53	51	53	50	36	35	39	44	(n = 361)
**Normal Joint**	10 (19)	12 (23)	14 (26)	8 (16)	5 (14)	4 (11)	14 (36)	15 (34)	**82(23)**
**Osteophytes**									
Absence	13 (25)	14 (28)	15 (28)	8 (16)	8 (22)	5 (14)	15 (39)	17 (39)	**95 (26)**
Presence	40 (75)	37 (72)	38 (72)	42 (84)	28 (78)	30 (86)	24 (61)	27 (61)	**266 (74)**
• Grade 1	5	3	8	8	2	3	5	2	**36**
• Grade 2	22	16	18	23	12	11	11	13	**126**
• Grade 3	13	18	12	11	14	16	8	12	**104**
**Erosions**									
Absence	27 (51)	16 (31)	26 (49)	25 (50)	8 (22)	9 (26)	20 (51)	24 (55)	**155 (43)**
Presence	26 (49)	35 (69)	27 (51)	25 (50)	28 (78)	26 (74)	19 (49)	20 (45)	**206 (57)**
• Grade 1	7	11	7	7	8	7	5	3	**55**
• Grade 2	6	10	10	10	8	3	5	7	**59**
• Grade 3	13	14	10	8	12	16	9	10	**92**
**BMLs**									
Presence	10 (19)	12 (24)	7 (13)	6 (12)	6 (17)	3 (9)	3 (8)	4 (9)	**51 (14)**
**Synovitis**									
Presence	7 (13)	10 (20)	6 (11)	2 (4)	1 (3)	0 (0)	3 (8)	0 (0)	**29 (8)**
**Destruction**									
Presence	12 (23)	11 (22)	8 (15)	6 (12)	14 (39)	16 (46)	8 (21)	12 (27)	**87 (24)**

The number (%) of affected joints is presented—BMLs, bone marrow lesions

* osteoarthritis: presence of a one abnormal feature.

### Concordance between MRI and RX for structural damage

Among the 55 patients, 361 joints were assessed on both RX and MRI. MRI showed 82 normal joints versus 91 on RX. MRI showed a higher prevalence of joints with structural lesions than RX (77.3% versus 74.8%—p<0.001) (Table 4).

**Table 4:** The agreement between MRI features and RX for the interphalangeal joints of 2nd–5th finger.

MRI feature		N feature	O feature	E feature	E/O feature	D feature	Total
Verbruggen–Veys	N	31 (34.1%)	22 (24.2%)	7 (7.7%)	21 (23.1%)	10 (11.0%)	91 (25.2%)
	**S**	42 (28.4%)	35 (23.6%)	2 (1.4%)	53 (35.8%)	16 (10.8%)	**148 (41.0%)**
	**J**	0 (0.0%)	4 (30.8%)	1 (7.7%)	6 (46.2%)	2 (15.4%)	**13 (3.6%)**
	**E**	6 (8.2%)	5 (6.8%)	0 (0.0%)	15 (20.5%)	47 (64.4%)	**73 (20.2%)**
	**R**	3 (8.3%)	5 (13.9%)	2 (5.6%)	14 (38.9%)	12 (33.3%)	**36 (10.0%)**
**TOTAL**		**82 (22.7%)**	**71 (19.7%)**	**12 (3.3%)**	**109 (30.2%)**	**87 (24.1%)**	**361**

The chi-square test was used for data’s analysis (p value < 0.001).

For VV score: N–Normal joint; S–Stationary phase, J—Joint space phase, E–Erosive phase R–Remodelling phase.

For MRI: osteophytic feature (O), erosive feature (E), erosive and osteophytic lesion feature (E/O) and complete articular destruction (D).

The concordance for the diagnosis of a normal joint on both imaging methods was low (34%); the same was true for the diagnosis of structural features of the joints (Table 4). MRI showed 121 (30.5%) erosive joints (E and E/O) against 73 (20.2%) phase E lesions in VV score and only 15 were present in both imaging modalities.

### Concordance between synovitis, BMLs and structural lesions on both modalities

There was no increasing distribution of synovitis and BMLs depending on the severity of the RX lesions. Furthermore, no lesion was significantly associated with a VV radiographic stage. (Table 5)

**Table 5:** The agreement and the ORs (95% CI) between synovitis, BMLs and Verbruggen-Veys radiographic scoring system features of the interphalangeal joints of the 2nd–5th fingers.

Verbruggen–Veys		Phase N	Phase S	Phase J	Phase E	Phase R	Total
		(n = 91)	(n = 148)	(n = 13)	(n = 73)	(n = 36)	(n = 361)
**Synovitis**	**0**	82 (90.1%)	137 (92.6%)	11 (84.6%)	66 (90.4%)	36 (100%)	**332 (92.0%)**
	**1**	2 (9.9%)	11 (7.4%)	2 (15.4%)	7 (9.6%)	0 (0%)	**29 (8.0%)**
	**OR [95%CI]**	1.0	0.7 [0.3–1.8]	1.7 [0.3–8.7]	1.0 [0.36–2.7]	N/A*	
**BMLs**	**0**	80 (87.9%)	129 (87.2%)	11 (84.6%)	60 (82.2%)	30 (83.3%)	**310 (85.9%)**
	**1**	11 (12.1%)	19 (12.8%)	2 (15.4%)	13 (17.8%)	6 (16.7%)	**51 (14.1%)**
	**OR [95%CI]**	1.0	1.1 [0.5–2.4]	1.3 [0.3–6.8]	1.6 [0.7–3.8]	1.5 [0.5–4.3]	

Binary logistic regression analyses were performed to describe and study the association.

Joints without osteoarthritis (feature N) served as the reference. NA, not applicable—BMLs, bone marrow lesions—OR, odds ratio; 95%CI, confidence interval [–].

* any patient has Erosive Feature and BMLs.

The prevalence of synovitis and BMLs tended to increase with increasing structural damage severity (Table 6). Patients with feature D presented more synovitis and BMLs than did patients with other features. For synovitis, 29 joints (8.0%) presented intra-articular inflammation, with a lower prevalence in normal joints (3.4%) compared to feature D joints (41.4%). An association between the MRI structural features and synovitis prevalence was observed (p<0.001) (Table 6).

**Table 6:** The agreement and the ORs (95% CI) between MRI features and synovitis, BMLs of the interphalangeal joints of the 2nd–5th fingers.

Features		Feature N (n = 82)	Feature O (n = 71)	Feature E (n = 12)	Feature E/O (n = 109)	Feature D (n = 87)	Total (n = 361)
**Synovitis**	**0**	81 (24.4%)	67 (20.2%)	11 (3.3%)	98 (29.5%)	75 (22.6%)	**332 (92.0%)**
	**1**	1 (3.4%)	4 (13.8%)	1 (3.4%)	11 (37.9%)	12 (41.4%)	**29 (8.0%)**
	**OR [95%CI]**	1.0	3.9 [0.5–8.3]	7.4 [1.8 to 30.7]	8.4 [1.8–13.6]	13.7 [2.9–21.0]	
**BMLs**	**0**	81 (26.1%)	67 (21.6%)	12 (3.9%)	91 (29.4%)	59 (19.0%)	**310 (85.9%)**
	**1**	1 (2.0%)	4 (7.8%)	0 (0.0%)	18 (35.3%)	28 (54.9%)	**51 (14.1%)**
	**OR [95%CI]**	1.0	3.9 [0.6–8.9]	N/A*	15.7 [3.2–23.5]	38.5 [9.5–57.0]	

Binary logistic regression analyses were performed to describe and study the association.

Joints without osteoarthritis (feature N) served as the reference. NA, not applicable—BMLs, bone marrow lesions—OR, odds ratio; 95%CI, confidence interval [–].

* any patient has Erosive Feature and BMLs.

For MRI: osteophytic feature (O), erosive feature (E), erosive and osteophytic lesion feature (E/O) and complete articular destruction (D).

Fifty-one joints (14.1%) presented BMLs. The association between MRI structural features and BMLs was significant (p<0.001). Only one normal joint presented BMLs (2%). As for synovitis, BMLs increased structural damage severity. More than half of the joints with BMLs corresponded to feature D joints (Table 6).

Analysis of the relationship between synovitis, BMLs and the anatomical VV score did not reach statistical significance according to the different sub-scores (synovitis and BMLs Chi-square tests yielded p = 0.30 and p = 0.83, respectively).

### Synovitis, BMLs according to MRI structural features

Joints with E/O and D presented significantly more synovitis and BMLs than did normal joints (Table 6). For E/O and D, the odds ratios for synovitis were 8.4 (95% CI 1.8 to 13.6; p = 0.017) and 13.7 (95% CI 2.9 to 21.0; p = 0.001), respectively. The same observation was made for BMLs: E/O: OR 15.7 (95% CI 3.2 to 23.5; p<0.001) and D: OR 38.5 (95% CI 9.5 to 57.0; p<0.001).

## Discussion

To the best of our knowledge, this is the first study aiming to compare structural damage according to the anatomical VV score to synovitis, BMLs and structural 0.3 T MRI features in a large sample of patients with EHOA. This study presented a new qualitative approach to EHOA lesions through the anatomical VV score and an MRI assessment based on 5 different features: N: normal joint, O: joint with only osteophytic lesion, E: joint with only erosive lesion, E/O: joint with osteophyte and erosion and D: destroyed joint. This differs from previous studies in which each elementary lesion (e.g., osteophyte, erosion, joint space narrowing, bone attrition) on MRI and RX is graded independently in terms of severity, and then the lesions are compared to each other [21–23,27].

As described by other authors using other RX scoring, we confirmed that MRI detects more structural lesions, e.g., osteophytes, erosion and destruction, than RX at the joint level, whereas only 70% of the joints available on RX were scored on MRI. As previously described, we confirmed a low concordance between MRI and RX with regard to the assessment of normal and abnormal joints [23]. In particular, our study described more erosive lesions on MRI than on RX.

Synovitis and BMLs were not associated with the grading of joint structural lesion on RX in different phases of VV score. Interestingly, we showed that synovitis and BMLs were mainly observed in joints with destruction but also in where both erosion and osteophyte were present together (E/O and D).

### Radiography and MRI structural damage

Our results are in agreement with those of previous studies [29,30]. On MRI, structural lesions also affected the DIP joints. In order, the joints most commonly affected were the 3rd DIPs (89%), followed by the 2nd DIPs (86%) and the 2nd PIPs (81%) (Table 3); the results were in agreement with those of previous studies [23,31].

At the joint level, 74% (n = 266) of joints were osteophytic and 57% (n = 206) were erosion. In 106 patients with HOA explored with 1T MRI, Haugen et al. found the same level of structural lesions with 86.4% and 50.8% joints with osteophytic and erosive lesions, respectively [23]. That study was conducted in HOA patients with a large majority of EHOA (%). On 1.5T MRI, Ramonda et al. also found a similar prevalence for osteophytes (73.9%) and erosions (51.1%) in a population exclusively with HOA [31]. Data from the literature also showed more structural lesions on MRI than RX using the Kellgren and Lawrence radiographic scoring system [23]. The superiority of MRI can be explained by a better contrast resolution between joint structures and by multiplanar acquisition which permitted to confirm structural lesions in at least two planes. While RX has the highest spatial resolution imaging (100μm^2^), this technique is limited by its contrast resolution being restricted to calcified joint structures and to its acquisition in two dimensions.

### Synovitis and BMLs

Synovitis and BMLs are observed in EHOA when severe structural damage was graded according to KL scoring method [23]. Haugen et al. demonstrated in HOA that BML prevalence increased with increasing structural damage severity, with odds ratios ranging from 2.6 (95% CI 1.2 to 5.4) to 11 (95% CI 5.5 to 21) for grade 1 and grades 3–4, respectively, with normal joints as the reference group [23]. However, the prevalence of synovitis was less associated with structural damage, with ORs ranging from 1.5 (95% 1.0 to 2.1) for Kellgren and Lawrence grade 1 to 1.4 (95% 1.0 to 2.2)) for Kellgren and Lawrence grade 4. In our patients, 51 and 29 joints exhibited BMLs (14%) and synovitis (8%), respectively. The prevalence of BMLs in our patients is similar to that in Haugen et al. (12.8%), whereas the prevalence of synovitis is lower in our study (8% versus 65.7%) [23]. In EHOA, Ramonda et al. reported higher prevalence of synovitis (39.8%) and BML (20.5–23.9%) [31]. This difference can be explained by a lower spatial resolution (0.3 Tesla MRI scanner), thus limiting detection only to the most severe synovitis. In fact, the prevalence of synovitis in the Haugen et al. study (1.0 Tesla MRI scanner) diminished to 22.3% when considering only the most severe grade (over 2) [21]. Compared to ultrasound, synovitis prevalence at 0.3T (8%) was quite similar to those reported by Kortekaas et al. [32].

In a pilot study, Tan et al. investigated differences in MRI features between patients suffering from hand psoriatic arthritis (PsA) and patients suffering from HOA[33]. They found similar features on MRI for both diseases, with more inflammatory lesions in the collateral ligaments and extensor tendons and more enthesis in DIP joints for PsA patients [33]. However, this study was performed on a 1.5 T MRI scanner with a dedicated coil protocol permitting the acquisition of a very high spatial resolution (160–200μm) images [34]. In our study, infra millimetric erosion, tenosynovitis, and BMLs localized near ligament insertion could not be visualized on 0.3 T machine with lower spatial resolution. For this reason, we decided to grade BML and synovitis joint destruction in a binary manner. Using this MRI scoring method, we demonstrated the ability to score structural features and synovitis, BMLs on a 0.3T scanner with a method that was highly reproducible between two readers (junior and senior) (K>0.75).

### Limits

The small number of patients included is one of the limitations of this study. Detailed joint analysis shows that only 70% and 94% of the DIPs and PIPs available on RX were scored by MRI. Thus, the absolute number of MRI lesions (structural lesions, synovitis and BMLs) is probably underestimated, limiting the statistical power of the analysis for the DIP joints but not for the PIP joints. We knew from Haugen et al. that HOA lesions visible on RX are associated with tenderness at the joint level [1]. Moreover, the development of an erosive RX lesion is an independent factor predicting tenderness joints. Clinical, radiographical and MRI were presented only for the dominant hand. Information concerning clinical data was obtained at the hand level and not at the joint level of the dominant hand. As a result, it was possible to establish a direct comparison at joint level between radiography and MRI, but not between clinical features and imaging modalities.

In conclusion, this study demonstrates that a 0.3T MRI scanner is highly reproducible and able to depict synovitis, BMLs and structural lesions in a sample of patients with EHOA. We showed that Synovitis and BMLs on MRI were more common in joints with severe joint lesions and especially in destroyed joint. MRI was also able to detect significantly more erosions than conventional radiographs according to VV. Data are currently being collected to evaluate the responsiveness of MRI features at one year of follow-up.
